# Evaluation of neuropathic pain with diverse pathophysiologies in childhood cancers

**DOI:** 10.14744/nci.2021.60133

**Published:** 2022-07-06

**Authors:** Hilal Susam Sen, Tugce Aksu Uzunhan

**Affiliations:** 1 Department of Pediatric Oncology, University of Health Sciences, Okmeydani Training and Research Hospital, Istanbul, Turkiye; 2 Department of Pediatric Neurology, University of Health Sciences, Okmeydani Training and Research Hospital, Istanbul, Turkiye

**Keywords:** Cancer, child, neuropathic pain, osteosarcoma, vincristine

## Abstract

**OBJECTIVE:**

Neuropathic pain (NP) is caused by damage or disease affecting the somatosensory nervous system. The aim of this study was to evaluate the clinical characteristics, pathophysiologies, and treatments applied in pediatric cancer patients with NP.

**METHODS:**

Patients with cancer having NP between 5 and 18 years of age who were followed up in the pediatric oncology clinic of Okmeydani Training and Research Hospital between January 2015 and April 2019 were included in this study. NP was described as tingling, burning, and stinging. Patients with acute lymphoblastic leukemia and brain tumors were excluded from the study. A number of pediatric cancer patients were also recorded. Patients’ age, gender, cancer diagnosis, NP characteristics and causes, treatments, and response to those treatments were investigated retrospectively and groups of NP according to their pathophysiological mechanism were established.

**RESULTS:**

NP was found in 26 (16%) of 160 patients followed up for childhood cancers. The average age was 11.8±4 years. Ten of the patients (38.4%) were female, and 16 (61.5%) were male. Osteosarcoma was the most common diagnosis in 10 (38%) patients. The most common cause of NP was compression of a nerve/root/spinal cord in 9 (35%) patients and the second most common was related with limb-sparing surgery. NP was found to be associated with chemotherapy (CT) in 5 (19%) patients, mostly with vincristine. Gabapentin was administered in a total of 22 (85%) patients for treatment. Opioid administration was more common as the disease stage progressed (p<0.05). A good or partial response to treatment was achieved in 19 (73%) patients.

**CONCLUSION:**

NP can occur in childhood cancers and is related to the cancer itself, CT, surgical treatment, and disseminated disease. Although there is no standard protocol, gabapentin and, for advanced-stage patients, opioids are the most commonly used treatment options.

## Highlight key points

- There is no standard treatment for NP in patients with childhood cancer.
- We found that in the treatment of NP, the frequency of opioid use increased significantly as the stage of cancer increased independently of the type of cancer.
- A single gabapentin or a combination treatment can be useful for the main treatment in patients with NP.

Children with cancer may experience various types of pain at the time of diagnosis, during chemotherapy (CT), and as the disease progresses. Different pain mechanisms may involve somatic, visceral, and neural tissues [1]. Neuropathic pain (NP) is defined by the International Association for the Study of Pain Special Interest Group on NP as “pain arising as a direct consequence of a lesion or disease affecting the somatosensory system” [2]. This type of pain is not well described in children but has been extensively researched in adults [2–4]. One reason for the lack of pediatric research is that children with NP may have age-related developmental limitations in terms of their ability to differentiate NP descriptors (i.e., burning, tingling, pins, and needles) from nociceptive pain descriptors (i.e., aching, pressure, and dull pain) and to express the specific characteristics of the pain [5]. The literature describing NP in pediatric oncology is commonly represented by case series, whereas stronger evidence from prospective trials or retrospective studies is limited. In the literature, different pathophysiological conditions have been evaluated in separate studies [1, 6, 7]. A small number of studies combined all of these pathophysiologies [8]. Therefore, in this study, our aim was to evaluate the clinical characteristics, NP pathophysiologies, and treatments of patients with childhood non-central nervous system (CNS) cancer who had NP in a pediatric oncology clinic.

## MATERIALS AND METHODS

Childhood cancer patients with NP between 5 and 18 years of age followed up in the pediatric oncology clinic of the Okmeydani Training and Research Hospital between January 2015 and April 2019 were included in the study. We do not have a palliative care department or any special pain department for pediatric oncology patients, so pediatric patients with NP and cancer could only be reached through the pediatric oncology clinic to be included in the study.

To evaluate the frequency of NP in our oncology clinic, the total number of pediatric cancer patients with similar characteristics as those investigated in this paper was recorded during the study. Pain in the form of tingling, burning, and stinging was evaluated as NP. Patients with acute lymphoblastic leukemia (ALL) were excluded from the study because those patients were treated in pediatric hematology clinic in our hospital. And also, CNS tumors were excluded from the study. Other exclusion criteria were prior treatment for a different cancer, having a neuromuscular disease associated with NP, and having an intellectual disability that would make it difficult to describe NP. Information about patients’ age, gender, cancer diagnosis, cancer stages, diagnostic features, laboratory investigations, NP characteristics and causes, treatments, and response to those treatments was recorded retrospectively. Five major pathophysiological groups were identified: After-amputation, after-limb-sparing surgery, associated with CT/radiotherapy (RT), nerve/root/spinal cord compression by a tumor, and disseminated disease. If NP occurred after amputation/limb-sparing surgery, it was grouped into these two categories. NP was considered to be CT related if CT had been administered within the previous 7 days. Nerve/root/spinal cord compression by a tumor is the responsible pathophysiology when such involvement is shown by the radiological methods and no other cause is possible. When there is widespread metastatic malignant neoplastic disease with NP and no other etiology can be detected, the NP cause was labeled as disseminated disease.

For our statistical analysis, because the most common diagnosis was bone tumors, the diagnoses were divided into either bone tumors or non-bone tumors. The different stages were further separated into two groups: Stage-2-and-below and Stage-3-and-above. The drugs administered in the treatment of NP were determined according to the physician’s initiative and the clinical status of the patient. The response to treatment was classified into four groups: Complete response, intermediate response, poor response, and no response. The patients with a complete response were those who had a complete or almost complete disappearance of the pain with treatments. Those participants with an intermediate response experienced a significant reduction in pain; patients with a poor response had inadequate pain relief, and those in the no response group had pain that did not diminish at all. After the first drug treatment, the second or third drug treatments were given to patients in the poor response and no response groups. The response status was defined as the final status of the patient after all treatments. Informed consent was obtained from the patients and their relatives before treatment. The Okmeydani Training and Research Hospital Ethics Committee of our institution approved the study (2019/1322).

### Statistical Analysis

Descriptive statistical analyses were performed to determine the demographic data, clinical data, and treatment of the patients. A Chi-square test was used for categorical variables. All statistical tests were performed using the Statistical Package for the Social Sciences, Chicago, USA, version 21.0.

## RESULTS

In total, 160 patients were observed in the pediatric oncology clinic during the study period due to non-ALL and non-CNS cancers; 26 of them (16%) had NP. Ten (38.4%) of the patients were female, and 16 (61.5%) were male. The average age was 11.8±4 years; the median age was 12.5, while the youngest patient was 5 and the oldest was 18. Osteosarcoma was the most common diagnosis in 10 (38%) patients, followed by Ewing’s sarcoma in 5 (19%) patients. Seven patients (27%) were in Stage 2, 9 (35%) were in Stage 3, 9 (35%) were in Stage 4, and 1 (3%) was in Stage 2 but not responding to CT. The most common cause of NP in 10 (38%) patients was compression of a nerve/root/spinal cord by a tumor. The pathophysiologies and the primary diagnoses are presented in Table 1.

**Table 1:** Distribution of causes of neuropathic pain by diagnosis

	Amputation/ phantom pain (n=3)	Limb-sparing surgery (n=5)	CT^1^/RT^2^ related (n=5)	Nerve/root/spinal cord compression (n=10)	Disseminated disease (n=3)
Osteosarcoma	3	4	1	1	1
Ewing’s sarcoma	–	–	–	4	1
Malignant mesenchymal tumor	–	–	–	3	–
Neuroblastoma	–	–	–	2	–
Non–Hodgkin lymphoma	–	–	1	–	–
Hodgkin lymphoma	–	–	1	–	–
Synovial sarcoma	–	1	–	–	–
Pleuropulmonary blastoma–	–	–	1	–	–
Nasopharyngeal carcinoma––	–	–	1	–	–
Pancreatic neuroendocrine tumor––	–	–	–	–	1

1 CT: Chemotherapy; ^2^RT: Radiotherapy.

Primary tumor localization was most commonly in the distal femur in osteosarcoma patients and in the iliac bone in Ewing’s sarcoma patients. All patients reported tingling, burning, and stinging pain. Twenty (77%) patients had hyperalgesia; 9 (35%) had allodynia, and all patients with allodynia also experienced hyperalgesia. All patients received CT. In five patients, NP was considered to be associated with CT, most commonly vincristine followed by cisplatin. Only one patient had received triple CT with brentuximab, vincristine, and cisplatin. Vincristine was withdrawn from treatment in one patient who underwent surgery for pleuropulmonary blastoma due to neuropathy, but the other patients completed their CT treatment. One patient with non-Hodgkin’s lymphoma had a diagnosis of ataxia telangiectasia confirmed by genetic analysis. The patient with Stage 2 Hodgkin’s lymphoma that did not respond to CT had undergone autologous stem cell transplantation followed by brentuximab vedotin. That patient had reported no symptoms of NP or neuropathy before treatment with brentuximab vedotin monotherapy. NP was treated with a single drug in 17 patients; 15 (58%) received gabapentin, one received fentanyl, and one received a nonsteroidal anti-inflammatory drug (NSAID) as the single agent. Gabapentin was administered in single or combination treatment in 22 (85%) patients. In nine patients, NSAIDs, gabapentin, fentanyl, and oxycodone combinations were used. NSAIDs, gabapentin, and fentanyl were combined in the treatment of a patient with Ewing’s sarcoma of the iliac bone. The pain of an operated osteosarcoma patient with phantom pain due to amputation decreased after prosthesis application. Only one patient with a malignant mesenchymal tumor had undergone physical therapy in addition to medical treatment with gabapentin. A complete response to treatment was observed in 7 (27%) patients, while 12 (46%) had an intermediate response, 2 (8%) experienced a poor response, and 5 (19%) did not respond. Nineteen (73%) patients had complete and intermediate responses, while 7 (27%) had poor responses or did not respond to treatment. Unresponsiveness was observed in two children with osteosarcomas, two children with Ewing’s sarcomas, and one child with a pancreas neuroendocrine tumor. Six patients did not survive: Three with osteosarcomas, one with Ewing’s sarcoma, one with operative neuroblastoma, and one with a pancreas neuroendocrine tumor.

There was no statistically significant relationship between the stage of the disease and the presence or absence of hyperalgesia (p>0.05), but there was a statistically significant relationship between the disease stage and opioid administration status (p<0.05) (Table 2). Accordingly, the rate of opioid use was significantly greater in the higher stages than in the lower stages. When the diagnoses were grouped as either bone tumors or non-bone tumors, there was no statistically significant relationship between opioid use and the diagnosis (p>0.05).

**Table 2:** Relationship between disease stage and other parameters

Variables	Stage of disease		χ^2^	p
	Stage 2 and below (%)	Stage 3 and above (%)		
Hyperalgesia			4.719	0.051
Yes (n=20)	20	80		
No (n=6)	67	33		
Opioid use			6.118	**0.023**
Yes (n=9)	0	100		
No (n=17)	47	53		

Gender, age, diagnosis, disease stage, primary tumor site, cause of NP, treatment of neuropathy, response to neuropathy treatment, and final status of the all patients are listed in Table 3.

**Table 3:** Data for all patients by diagnosis

Patient number	Gender	Age (year)	Diagnosis	Stage of disease	Primary tumor site	The cause of neuropathic pain	Neuropathy treatment	Response to neuropathy treatment	Final situation
1	M	8	Osteosarcoma	3	Distal femur	Limb-sparing surgery	Gabapentin	Intermediate response	Diseased alive
2	M	13	Osteosarcoma	2	Proximal tibia	Limb-sparing surgery	Gabapentin	Intermediate response	Diseased alive
3	M	10	Osteosarcoma	2	Distal femur	Limb-sparing surgery	Gabapentin	Complete response	Diseased alive
4	F	5	Osteosarcoma	2	Distal femur	Limb-sparing surgery	Gabapentin	Complete response	Diseased alive
5	M	16	Osteosarcoma	4	Distal femur	Amputation-phantom pain	Gabapentin Prosthesis	Complete response	Lost to follow-up
6	F	12	Osteosarcoma	4	Distal femur	Amputation-phantom pain	Oxycodone Fentanyl	No response	Exitus
7	M	7	Osteosarcoma	4	Distal femur	Amputation-phantom pain	Gabapentin Fentanyl	No response	Exitus
8	M	16	Osteosarcoma	4	Distal femur	Disseminated disease	NSAID Gabapentin Fentanyl	Poor response	Exitus
9	M	15	Osteosarcoma	4	Distal femur	Tumor root compression	Gabapentin	Complete response	Diseased alive
10	M	13	Osteosarcoma	2	Sacral bone	CT/RT cisplatin	Gabapentin	Intermediate response	Diseased alive
11	M	15	Ewing’s sarcoma (operated)	2	Iliac bone	Tumor root compression	Gabapentin	Intermediate response	Diseased alive
12	M	10	Ewing’s sarcoma (operated)	3	Iliac bone	Tumor root compression	NSAID Gabapentin Fentanyl	No response	Lost to follow up
13	F	13	Ewing’s sarcoma (operated)	4	Abdomen	Disseminated disease	Fentanyl Gabapentin	No response	Exitus
14	F	11	Ewing’s sarcoma	3	Sacral bone	Tumor root compression	Fentanyl	Complete response	Diseased alive
15	F	18	Ewing’s sarcoma	3	Radius proximal	Tumor root compression	Gabapentin	Intermediate response	Diseased alive
16	F	17	Malignant mesenchymal tumor (operated)	2	Soft ankle dorsal tissue	Tumor root compression	Gabapentin	Intermediate response	Diseased alive
17	M	16	Malignant mesenchymal tumor	3	Distal femur	Tumor root compression	Gabapentin	Intermediate response	Lost to follow-up
18	F	10	Malignant mesenchymal tumor	3	Iliac region soft tissue	Tumor root compression	Gabapentin physiotherapy	Complete response	Diseased alive
19	M	3	Neuroblastoma (operated)	4	Abdomen	Spinal cord compression	Fentanyl Gabapentin	Poor response	Exitus
20	M	4	Neuroblastoma	4	Posterior mediastinum	Tumor spinal cord compression	Gabapentin Fentanyl	Intermediate response	Lost to follow-up
21	M	11	Non-Hodgkin’s lymphoma	3	Mediastinum	CT Vincristine	Gabapentin	Intermediate response	Diseased alive
22	M	13	Hodgkin’s lymphoma	2	Cervix	CT Vincristine Brentuximab vedotin Cisplatin	Gabapentin	Intermediate response	Diseased alive
23	M	16	Synovial sarcoma (operated)	3	Distal femur	Limb-sparing surgery	NSAID Gabapentin	Complete response	Diseased alive
24	F	3	Pleuropulmonary blastoma (operated)	3	Mediastinum	CT Vincristine	NSAID Vincristine discontinued	Intermediate response	Diseased alive
25	F	12	Nasopharyngeal carcinoma	2	Rhinopharynx	CT Cisplatin Docetaxel	Gabapentin	Intermediate	Diseased alive
26	F	14	Pancreatic neuroendocrine tumor	4	Abdomen	Disseminated disease	Oxycodone Fentanyl	response No response	Exitus

## DISCUSSION

In our study, we found that the incidence of NP in non-CNS childhood cancers in our pediatric oncology clinic was 16% excluding leukemia. There are a few studies about NP frequency in the literature; in one of them, patients admitted to the pediatric oncology pain service for NP were evaluated, and the frequency of NP was found to be 17% [8]. Difficulties in identifying NP in childhood cancers have resulted in the emergence of both qualitative and quantitative tests. Quantitative sensory testing is effective in the diagnosis of NP; however, because it requires trained personnel and expensive equipment, its widespread use in clinical settings is limited [9]. The only clinical assessment tool used to score CT-induced peripheral neuropathy is the pediatric modified total neuropathy scale for 5–18 years old. In this scoring methodology, there are questions regarding sensory, motor, and autonomic symptoms and physical examination findings [10]. Due to our study’s retrospective nature, we could not score NP. Despite all efforts, there is no scoring method to reliably identify NP in children under 5 years old [11]. Therefore, we did not include any children younger than 5 years old since NP could not be clearly defined in this age group.

In our study, the most common type of cancer was osteosarcoma in 10 patients; 70% of these patients experienced operation-related pain. Approximately half of the patients underwent limb-sparing surgery, and the other half had amputation. The pathophysiological cause of NP in the remaining cancer patients was determined as CT/RT related, due to tumor compression of a nerve/root/spinal cord, or the result of disseminated disease. The priority in surgical treatment is, if possible, the removal of the tumor by limb-sparing surgery and reconstruction with an endoprosthesis or allograft. In cases where a limb-sparing procedure is not feasible, an amputation is recommended.

In the one and only prospective study evaluating NP after surgical treatment in pediatric osteosarcoma, it was observed that 81% of patients had NP [6]. In fact, NP occurs following 10–50% of common operations and is related to inflammation, stretching, contusion, and transection of nerves [12]. Phantom limb pain, a type of pain associated with surgical amputations that occur in the removed extremity, is very well known in the literature. About 60–90% of amputees suffer from phantom pain at some time in their lives [13]. In a prospective osteosarcoma study, no difference was found between amputation and limb-sparing surgery in terms of the frequency of NP, duration of treatment, and treatment doses. In our study, two patients with Stage 4 osteosarcoma who underwent amputation did not survive, and the other three patients who had an amputation did not respond or had a poor response to their treatment. In contrast, patients who underwent limb-sparing surgery had complete and intermediate responses to NP treatment. This result may be due to the fact that limb-sparing surgery cannot be performed in patients who already have progressive, intractable disease in the affected extremity.

Among the chemotherapeutic agents, as many as 50–90% of patients treated with platinum compounds, such as cisplatin and carboplatin, develop peripheral neuropathy, whereas with vinca alkaloids, the rate is 50% [14]. In a retrospective study, peripheral neuropathy with vincristine has been reported in 174 of 498 children with ALL [1]. In our study, vincristine was the most common cause of neuropathy/NP related to CT followed by cisplatin. Another agent associated with neuropathy is a relatively new chemotherapeutic approved for the treatment of patients with relapse/resistant Hodgkin’s lymphoma: Brentuximab vedotin. Brentuximab vedotin is an agent that acts by secreting conjugated microtubule-disrupting agent monomethyl auristatin E (MMAE) against CD30 cancer cells. Up to 73% of patients have neuropathy side effects, resulting in dose reduction or discontinuation of treatment [15]. The neuropathy is reported to be reversible after discontinuation of treatment. In our study, peripheral neuropathy and NP were observed in one patient after brentuximab vedotin, but no change in the treatment protocol was required; while the patient treated with gabapentin benefited. Anti-GD2 antibodies, another biological agent used in the treatment of disseminated neuroblastoma, may cause peripheral neuropathy in childhood [16]. In our study, in two patients with neuroblastoma, the cause of NP was spinal cord compression by the tumor.

In the treatment of pain in children and adolescents, the first step is NSAIDs [17]; when it comes to NP, α2δ agonists (gabapentin and pregabalin) are the first-line interventions. Historically, tricyclic antidepressants and serotonin norepinephrine reuptake inhibitors were the most preferred agents [3]. In patients who do not respond to first-line treatments for NP, opioids are administered after trying all other treatment options. This stepwise approach can be attributed to the undesirable side effects related to opioids as well as the development of opioid tolerance, opioid hyperalgesia, and addiction [11]. In our study, we found that in the treatment of NP, the frequency of opioid use increased significantly as the stage of cancer increased independently of the type of cancer. Disseminated disease and advanced stage patients were more resistant to treatment and required drugs with more potential side effects. Case reports have indicated that methadone, ketamine, and lidocaine treatments in childhood cancer are also effective in treating NP caused by different pathophysiologies [18, 19]. In our study, for a small number of patients, NSAIDs were used in case the pain was a visceral pain. For the main treatment, a single gabapentin or a combination treatment was administered to 86% of the patients. In addition to pharmacological therapies for NP, non-pharmacological interventions, such as physiotherapy, should be applied whenever possible [10]. Physical therapy was performed in only one patient with a malignant mesenchymal tumor with NP due to spinal root compression.

The key limitation of our study was its retrospective design. Because of its retrospective character, the pain level could not be evaluated, the duration of pain was not clear, and the treatment doses could not be included in the study. We also may have missed a few children harboring NP during cancer treatment because the study design was not prospective. However, in our study, we evaluated the clinical characteristics, treatment types, and treatment responses of children with cancer and also NP with different pathophysiologies followed by a single center.

## Conclusion

While 20–40% of adults experience NP during cancer [20], the incidence of cancer-related NP in childhood is unknown.[4] We examined the prevalence of NP in all children with various non-CNS solid tumors in our pediatric oncology clinic. Our study is one of a few to have evaluated NP associated with childhood cancers with the specific aforementioned features. With the guidance of further multicenter prospective studies, it may be possible to establish standard treatment protocols for the diagnosis and treatment of NP in childhood cancers.
